# A Rapid Intraoperative Parathyroid Hormone Assay Based on the Immune Colloidal Gold Technique for Parathyroid Identification in Thyroid Surgery

**DOI:** 10.3389/fendo.2020.594745

**Published:** 2021-04-22

**Authors:** Wenfei Xia, Jinjun Zhang, Wenzhuang Shen, Zhi Zhu, Zhifang Yang, Xingrui Li

**Affiliations:** ^1^ Department of Thyroid and Breast Surgery, Tongji Hospital, Tongji Medical College, Huazhong University of Science and Technology, Wuhan, China; ^2^ Department of Reproductive Medicine, Tongji Hospital, Tongji Medical College, Huazhong University of Science and Technology, Wuhan, China

**Keywords:** immune colloidal gold technique, hypoparathyroidism, parathyroid hormone, electrochemiluminescence immunoassay, parathyroid gland

## Abstract

**Objective:**

A novel immunochromatographic test strip method was developed to detect tissue parathyroid hormone (PTH) using the immune colloidal gold technique (ICGT). The accuracy and application value of this method for intraoperative parathyroid identification were evaluated.

**Methods:**

Serum samples were collected to measure PTH by both ICGT and electrochemiluminescence immunoassay (ECLIA). Patients who underwent unilateral and total thyroidectomy were enrolled to evaluate the feasibility and clinical efficacy of rapid intraoperative identification of parathyroid glands *via* PTH determination using ICGT. Two sample preparation methods, fine needle aspiration (FNA) and tissue block homogenate (TBH), were used for PTH-ICGT analysis.

**Results:**

Bablok analysis showed a linear relationship between the serum PTH measurements obtained by ICGT and ECLIA. Non-parathyroid tissues had much lower PTH concentrations (14.8 ± 2.1 pg/ml, n = 97) detected by ICGT, compared to the parathyroid gland tissues (955.3 ± 16.1 pg/ml, n = 79; P < 0.0001), With biopsy results as the standard, ICGT showed higher diagnosis rates as compared with direct visual inspection, for identifying both parathyroid glands (97.4 vs. 78.2%) and non-parathyroid tissues (100 vs. 68.9%). The cut-off values for parathyroid identification by FNA and TBH methods were 63.99 and 136.30 pg/ml, respectively. The detection time was 2 min by TBH method for *in vitro* tissue detection and 6 min by FNA method for *in situ* tissue detection, both of which were faster than traditional intraoperative cryopathological examination (usually >30 min). Intraoperative application of ICGT method was associated with higher postoperative serum calcium and blood PTH levels at 1 and 3 months as well as a lower incidence of postoperative transient hypocalcemia, as compared with direct visual inspection.

**Conclusion:**

PTH-ICGT assay shows high potential as a rapid, novel alternative for intraoperative parathyroid identification.

## Introduction

Hypoparathyroidism is an abnormal condition characterized by low levels of parathyroid hormone (PTH) (1). The most common cause for hypoparathyroidism is accidental removal or injury of the parathyroid glands during thyroid or neck surgery (2). Up to 60% of patients who undergo total thyroidectomy experience transient hypoparathyroidism, manifested as hypocalcemia, within a few days after thyroid surgery (3). Although most patients with a low postoperative PTH recover parathyroid function quickly, approximately 4% of them develop a permanent hypoparathyroidism that persists beyond 6 months after surgery (3, 4). Thus, *in situ* preservation of the parathyroid glands by distinguishing them from surrounding structures is of significance.

Currently, intraoperative identification of the parathyroid glands largely depends on the experience of a surgeon, leading to a considerable risk of accidental parathyroidectomy. Several auxiliary techniques are used during thyroid surgery to minimize the probability of parathyroid removal and the subsequent development of postoperative hypoparathyroidism and hypocalcemia, such as carbon nanoparticle suspension negative imaging, visible staining by methylene blue or antiparathyroid antibody BB5 G1 conjugated to Cibacron blue, and gamma probe identification (5–9). Unfortunately, until now there are no reliable intraoperative methods to identify parathyroid glands. Carbon nanoparticle suspension negative imaging can help identify the parathyroid gland but is less effective in identifying fat from parathyroid tissue (5, 10). Intraoperative frozen sectioning for histological examination is useful in differentiating parathyroid tissue from other tissues and is selectively used to identify the presence of parathyroid tissue and to provide histological confirmation for potential parathyroid autotransplantation. However, this method has many disadvantages in the identification of parathyroid glands. First, histological examination requires sample dissected from *in situ* parathyroid, which would often lead to tissue damages. Second, this technique is time-consuming, usually exceeding 30 min, which may delay the operation time and also affect the vitality of the remaining parathyroid glands to be autotransplanted, even resulting in transplantation failure.

PTH is a polypeptide with 84 amino acids and a molecular weight of 9.5 kDa, and it is specifically secreted from and expressed in the parathyroid glands (11). Measuring the PTH level in biopsy samples has been shown to be highly reliable for identifying parathyroid tissue (12, 13). Electrochemiluminescence immunoassay (ECLIA) using commercially available reagents is a reliable method for the measurement of serum PTH level. However, this assay requires expensive equipment and specially trained technical personnel, and is mainly installed in hospital testing centers for high-throughput serum PTH testing. The delivery of a single sample of tissue eluate requires close coordination among multiple departments; moreover, the inspection process exceeds 30 min. Inspired by this method of identifying parathyroid glands, we have developed a PTH detection technique using ICGT for identification of parathyroid glands. The detection instrument used is portable (< 500 g) and can be placed in the operating room. The new PTH detection technique is quantitative, easy-operated, minimally invasive and time-saving as it works within a minimum of 2 min. Also, it is also suitable for both *in vitro* and *in vivo* identification of parathyroid glands.

The aim of this study was to verify the consistency between the ICGT method and a standardized ECLIA method for the measurement of serum PTH concentrations. Importantly, we analyzed the reliability and accuracy of the ICGT method in identifying parathyroid glands during thyroidectomy, and evaluated the clinical value of this method in improving the efficiency of intraoperative parathyroid recognition and reducing the incidence of postoperative hypoparathyroidism. We also explored the sensitivity and efficiency of ICGT for the detection of PTH when different sample collection techniques were used.

## Materials and Methods

**Figure 1:**
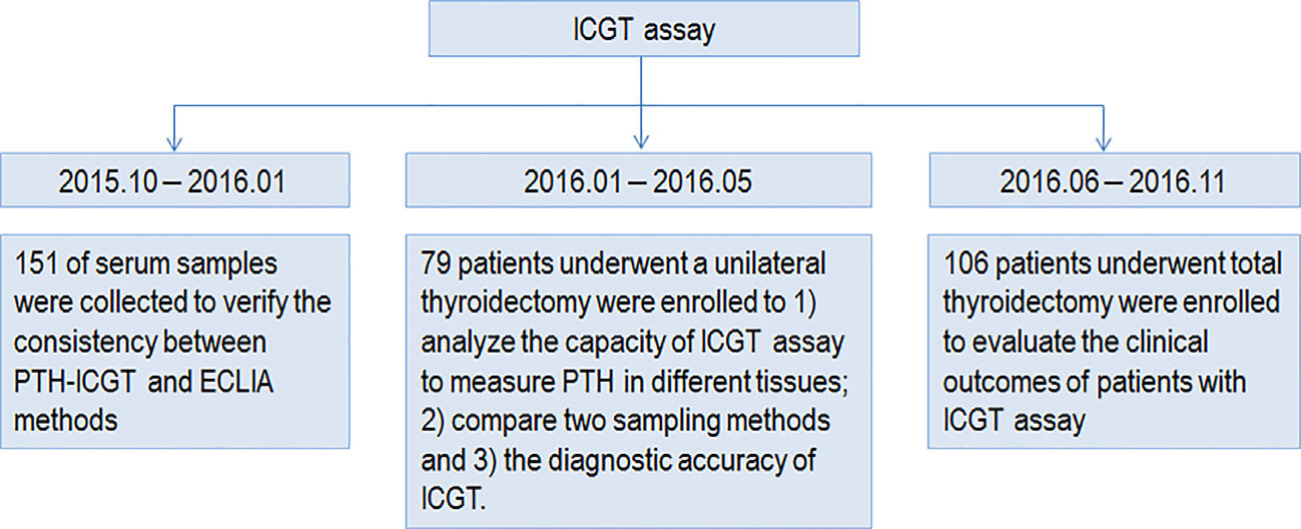
Study design and procedures.

The study was approved by the ethics committee of Huazhong University of Science and Technology (Wuhan, China). All procedures were conducted in compliance with the principles in the Declaration of Helsinki. Written informed consent was obtained from all individual participants included in the study. The study design and procedures are shown in **Figure 1**.

### Principle and Detection Procedures of Immune Colloidal Gold Technique Assay

**Figure 2:**
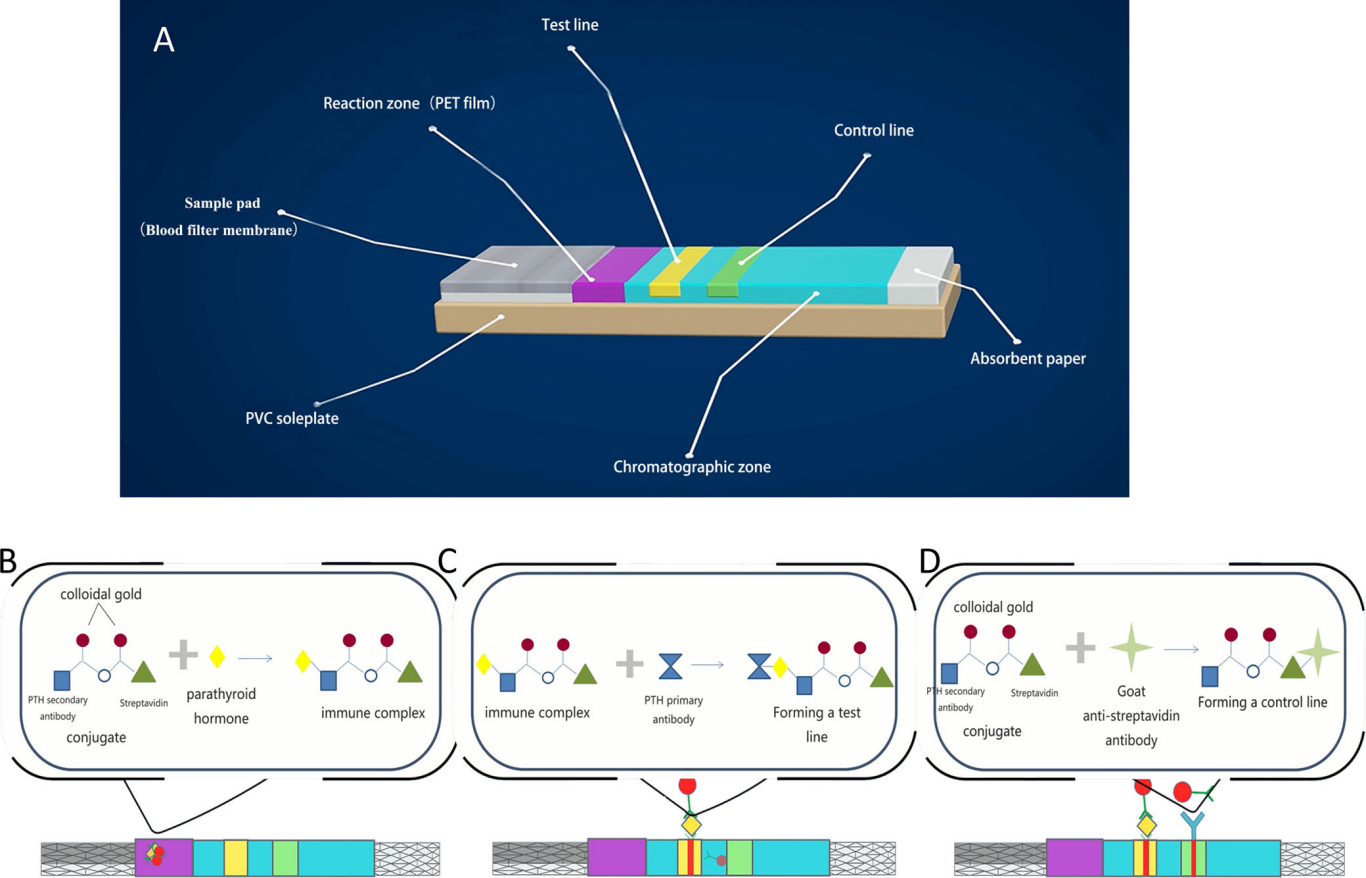
Principle and detection procedures of the PTH-ICGT assay. **(A)** Components of a test strip. **(B)** The PTH in the sample binds to the colloidal gold–antibody conjugates that are pre-coated on the conjugate pad to form an immune complex. **(C)** Through capillary action, colloidal gold-labeled complexes are captured by the solid-phase chromogenic agent (PTH antibody) located in the test zone on the membrane to form a pink or dark red ribbon. **(D)** The un-reacted complexes stagnate at the control zone to form a control line by binding to pre-coated anti-streptavidin antibody on the membrane. PVC, polyvinyl chloride; PET, polyethylene terephthalate.

The PTH-ICGT assay was developed by Bioda (China) and is a novel method utilizing the ICGT technique to determine the PTH level (**Figure 2**). As a 2-site immunoassay, the principle of the ICGT immunoassay is to use colloidal gold as a tracer marker in antigen-antibody reactions. The PTH detection kit consists of plastic boxes and test strips installed in the box. Using solid-phase chromogenic technique, the test strip is pre-coated with PTH primary antibody on the nitrocellulose membrane at the test zone (the binding site is at the C-terminal of the PTH peptide chain). The control zone is pre-coated with goat anti-streptavidin antibody. The chemical conjugate pad is coated with colloidal gold-labeled PTH secondary antibody (the binding site is at the 53–68 amino acid of the PTH peptide chain) and colloidal gold-labeled streptavidin conjugate.

**Figure 3:**
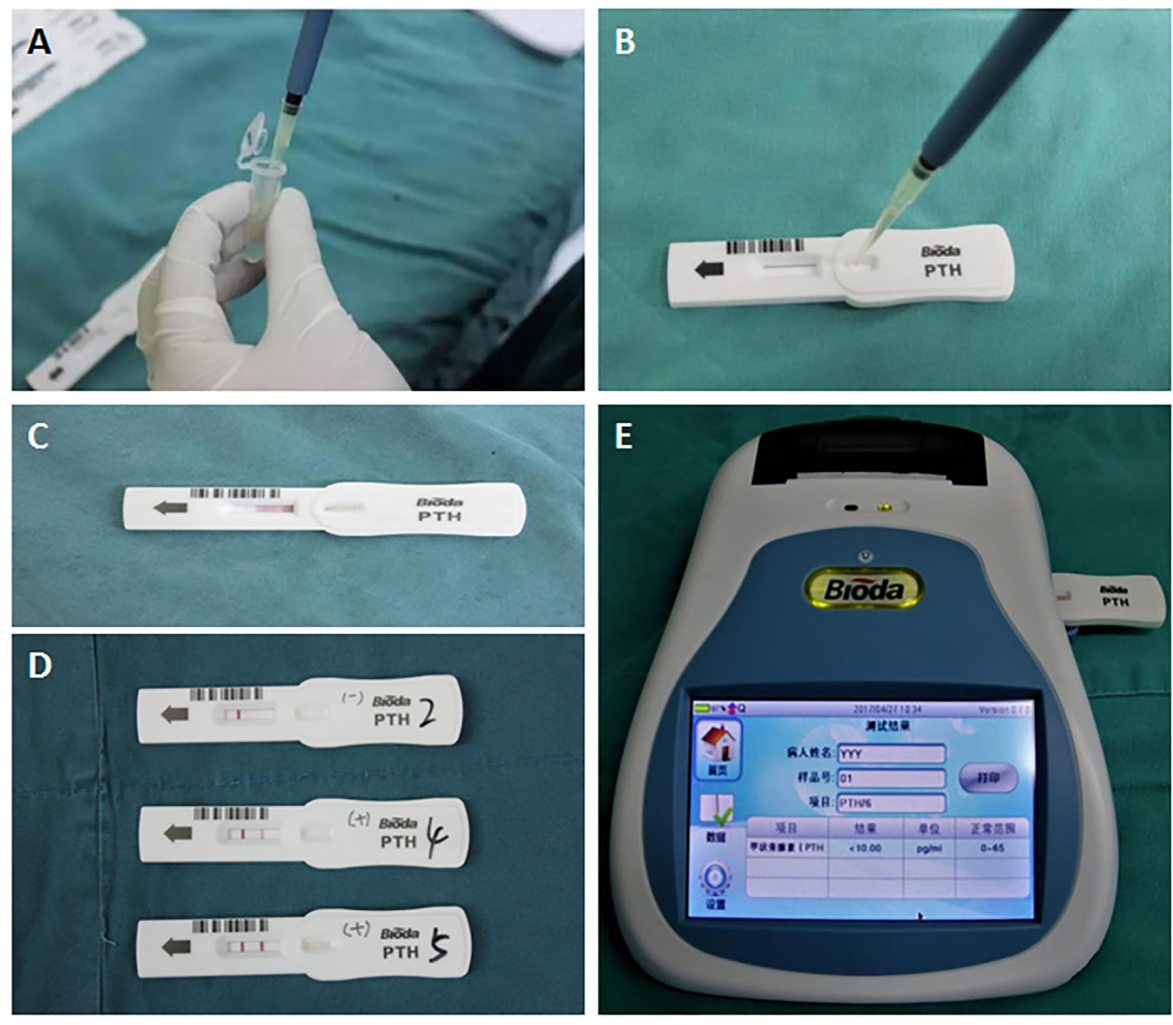
Detection procedure for the ICGT assay. **(A, B)** A serum or tissue homogenate sample was added into the hole of the strip. **(C)** Through capillary action, PTH in serum or tissue homogenate samples reacts with and bonds to the anti-PTH antibody. **(D)** The reaction in test zone on the membrane. Negative (blank, upper strip), weak positive (pink, medium strip), or strong positive (dark red, bottom strip). **(E)** PTH concentration on the test strip is quantitatively analyzed by a Bioda Immunity Ration Detector.

At the time of the detection, the sample (90 μl) to be tested is added into the hole of the strip. The PTH in the sample binds to the colloidal gold–antibody conjugates that are pre-coated on the conjugate pad to form an immune complex. Through capillary action, colloidal gold-labeled complexes are captured by the solid-phase chromogenic agent (PTH antibody) located in the test zone on the membrane to form a pink or dark red ribbon. The un-reacted complexes stagnate at the control zone to form a control line by binding to pre-coated anti-streptavidin antibody on the membrane. The degree of coloration of the ribbon is proportional to the PTH level in the sample tested within a certain range. The detection line and the quality control line are scanned and analyzed, and the parameters of the instrument are preset to calculate the PTH level in the blood. After incubation for 2 or 6 min, the PTH concentration on the test strip is quantitatively analyzed by a Bioda Immunity Ration Detector (Bioda Diagnostics, Wuhan, China), **Figure 3**. The detection range with ICGT assay was 10–1,000 pg/ml. The strip would display a negative result when the sample concentration was lower than the lowest detection limit (10 pg/ml); when the sample concentration was higher than 1,000 pg/ml, the instrument detection value showed >1,000 pg/ml. The detection coefficient of variation from the same batch of detection reagents was <15%. All measurements were carried out by the same technician.

### Sample Preparation for Immune Colloidal Gold Technique Assay

**Figure 4:**
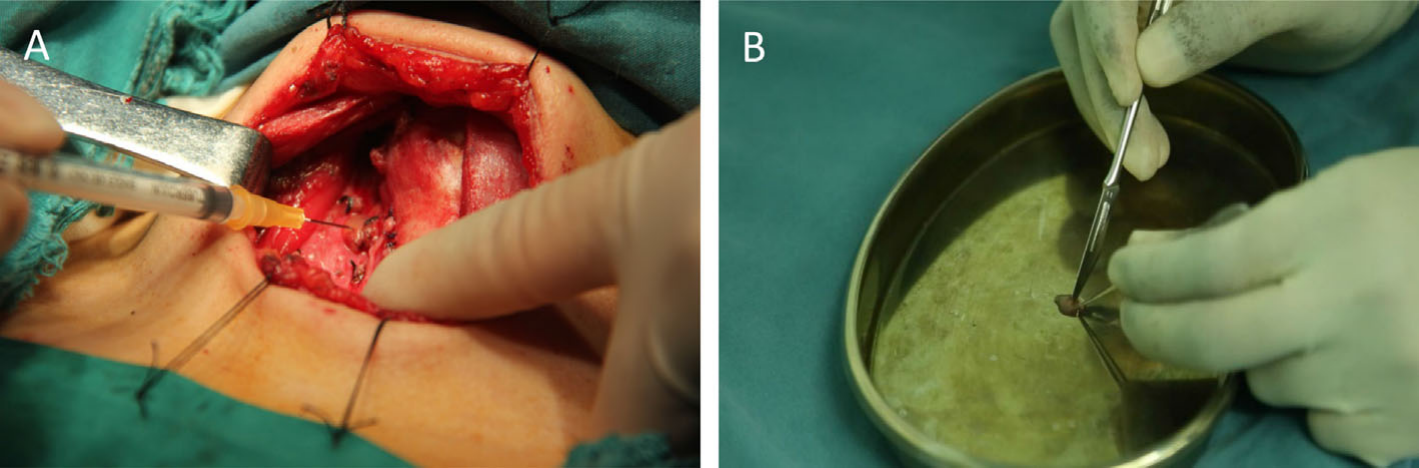
Different sample collection techniques for ICGT. **(A)** Fine-needle tissue aspiration (FNA). **(B)** Tissue homogenization (TBH).

Two sample preparation methods FNA and TBH were applied and compared for the PTH-ICGT assay (**Figure 4**). By the FNA method, the target tissue was aspirated by using a 1 ml syringe with negative pressure for three times, to obtain a small amount of tissue fluid, which was diluted in 1 ml of normal saline (0.9% w/v of NaCl). By the TBH method, 1 mm³ of the target tissue specimen was resected, cut into homogenate with a surgical blade, and then diluted in 1 ml of normal saline. The tissue eluates prepared using the above methods were assayed.

### Study Methods and Procedures

#### Part I

A preliminary study was performed to examine the consistency between the ICGT and ECLIA methods. PTH was detected in a total of 151 serum samples stored at –25°C after collection from patients who underwent a health examination at the Tongji Hospital of Tongji Medical College, Huazhong University of Science and Technology between October 2015 and January 2016. The serum PTH levels were measured by both the ICGT and classical ECLIA methods (ROCHE). The ECLIA determination, serving as a gold standard for measurement of PTH for comparison, was conducted with an automated ECLIA system (Roche Diagnostics GmbH, Mannheim, Germany).

#### Part II

Patients (n = 79) who underwent a unilateral thyroidectomy between January and May 2016 were enrolled. During the surgery, the distinction of parathyroid gland tissue from surrounding structures was first completed and recorded by direct visual inspection by surgeons with 4–6 years of surgical experience. A total of 176 tissue samples, including skeletal muscle, thyroid, fat, lymph node, and parathyroid, were collected from the patients. Before resection of tissue samples, a small amount of tissue fluid was taken by FNA for PTH determination by the ICGT assay. Then, 1-mm^3^ tissue samples were collected and divided into two halves. One half was used for frozen section examination, and from the other half, tissue homogenate was extracted and dissolved in 1 ml normal saline for PTH determination by the ICGT assay (TBH method). The identified parathyroid gland was preserved *in situ* or auto-grafted into muscle. With frozen section examination as the standard, 1) the PTH levels were compared among different tissues. 2) The cut-off value, sensitivity, and specificity of the sampling methods for intraoperative parathyroid identification by ICGT assay were analyzed, and the detection times of two sampling methods were also compared. 3) Also, the diagnostic accuracies of the ICGT assay (FNA, n = 139) versus direct visual inspection with the naked eye for intraoperative identification of parathyroid glands were compared.

#### Part III

Between June and November 2016, a total of 106 patients who underwent total thyroidectomy, including 21 males (19.8%) and 85 females (80.2%), aged 45.6 ± 17.3 years (range, 22–68 years), were enrolled. Using a random number table, the patients were randomly assigned to two groups to undergo intraoperative parathyroid identification via: detection of the tissue PTH level by ICGT assay (PTH-ICGT group, FNA, n = 53) or naked eye and experience of surgeons (control group, n = 53). In PTH-ICGT group, the ICGT method was used to assist the identification of parathyroid tissue during the operation. After detection and based on the results, the tissue was decided timely to retain *in situ* or underwent autologous transplantation. All operations were performed by the same team of surgeons.

The postoperative serum PTH concentrations were determined by Roche ECLIA, and serum calcium levels were regularly examined. The incidence of postoperative transient hypocalcemia, which was defined as a total serum calcium concentration ≤2.12 mmol/L (8.5 mg/dL) measured within 24 h after surgery, was recorded (14).

### Statistical Analysis

All statistical analyses were performed using SPSS16.0 software (SPSS Inc., Chicago, IL, USA) and GraphPad Prism software (version 6; GraphPad Software Inc., USA). Quantitative data are presented as mean ± standard deviation (SD). The categorical variables were expressed as an absolute number and percentage. Comparisons between two groups were performed using independent-samples t test. Categorical data were compared using chi-square or Fisher’s exact test. The consistency between the PTH-ICGT and ECLIA methods was examined by linear regression analysis. P <0.05 was considered statistically significant.

## Results

### Part I

#### Consistency Between Parathyroid Hormone-Immune Colloidal Gold Technique and Electrochemiluminescence Immunoassay Methods

**Figure 5:**
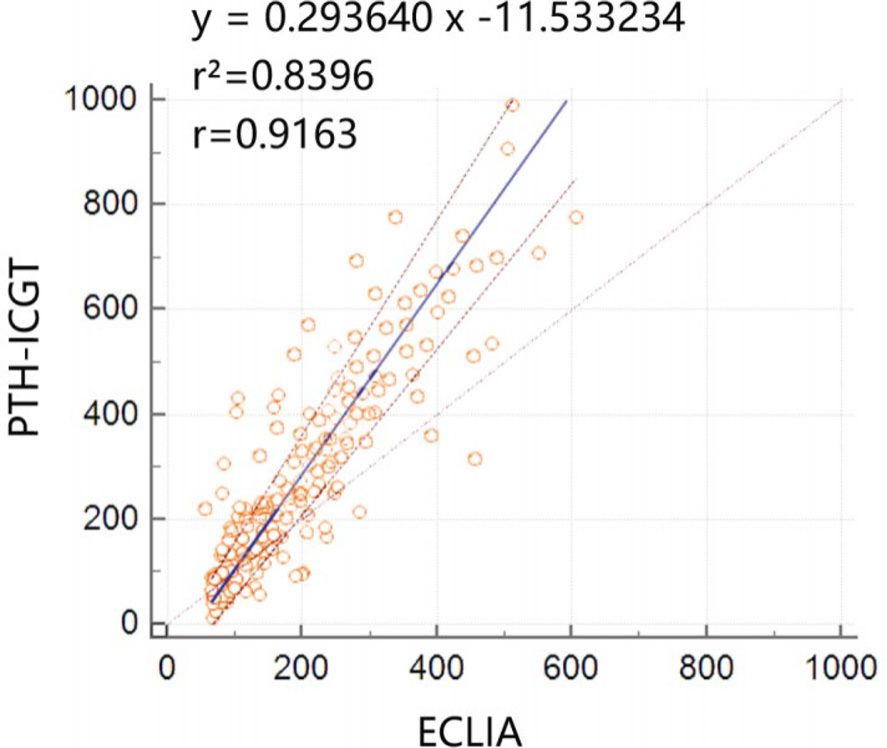
Consistency between the PTH-ICGT and ECLIA methods. The Passing Bablok analysis shows a linear relationship between the PTH measurement results detected by the PTH-ICGT and ECLIA methods, and the equation is y = 0.293640x –11.533234, where y is the PTH measurements (pg/ml) by the ICCT method, and x is the PTH measurements (pg/ml) by the ECLIA method. The correlation coefficient for consistency is 0.9163 (P < 0.0001).

A total of 151 serum samples were collected at the time of thyroid surgery to verify the consistency between PTH-ICGT and ECLIA methods. To minimize the bias caused by extreme values, PTH measurements >1,000 pg/ml, (n = 0) or <10 pg/ml, (n = 0) by either PTH-ICGT or ECLIA were excluded. The Passing Bablok analysis showed a linear relationship between the PTH measurement results detected by the PTH-ICGT and ECLIA methods, and the equation was as follows: y = 0.293640 x – 11.533234 (**Figure 5**), where y is the PTH measurement (pg/ml) by the ICCT method, and x is the PTH measurement (pg/ml) by the ECLIA method. The correlation coefficient for consistency was 0.9163 (P < 0.001), suggesting good consistency between the two methods.

### Part II

#### Comparison of Quantitative Parathyroid Hormone-Immune Colloidal Gold Technique Analysis for Different Tissues

**Figure 6:**
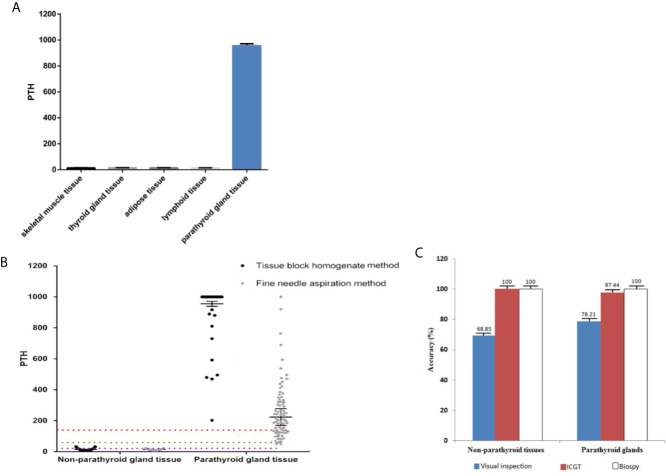
Comparison of quantitative ICGF analysis for different tissues. **(A)** PTH concentrations in parathyroid glands versus non-parathyroid tissues. **(B)** PTH levels of tissues collected by different sampling techniques. The box represents the median and upper and lower quartiles; the dotted lines below represent the cut-off values. **(C)** Diagnostic rate of the PTH-ICGT assay. With biopsy results as the standard, ICGT showed a higher diagnostic rate than direct visual inspection for identifying both parathyroid glands and non-parathyroid tissues.

A total of 79 patients, including 17 males (21.5%) and 62 females (78.5%), aged 40.6 ± 8.3 (range 28–65) years, were enrolled. The PTH levels detected by ICGT in different tissues or structures were compared in order to determine whether this quantitative PTH-ICGT assay can be used to differentiate parathyroid glands from other tissues. A total of 176 tissue samples were collected. As shown in **Figure 6A**, the PTH concentrations in non-parathyroid tissues of homogenate [overall (14.8 ± 2.1 pg/ml, n = 97); skeletal muscle (14.5 ± 1.5 pg/ml, n = 13), thyroid tissue (15.0 ± 1.3 pg/ml, n = 26), adipose tissue (15.3 ± 1.2 pg/ml, n = 36) and lymph nodes (14.0 ± 1.2 pg/ml, n = 22)] were significantly lower than those in the parathyroid gland (955.3 ± 16.1 pg/ml, n = 79, P < 0.0001). The data suggested that intraoperative tissue PTH levels allowed accurate distinction and detection of parathyroid tissue. Thus, it is feasible to distinguish parathyroid gland tissue from surrounding structures by PTH measurement using the PTH-ICGT assay.

### Comparison of Different Sampling Methods for Immune Colloidal Gold Technique-Parathyroid Hormone Detection

We compared the PTH levels in non-parathyroid and parathyroid tissues collected using different sampling techniques (**Table 1**). The PTH levels in parathyroid tissue were (955.3 ± 16.1) pg/ml and (224.0 ± 54.6) pg/ml when detected by ICGT in samples collected by the TBH and FNA methods, respectively (**Figure 6C**). The PTH levels in parathyroid gland were much higher than those in non-parathyroid tissues, irrespective of the sampling technique employed (TBH 15.3 ± 0.9 pg/ml and FNA 14.1 ± 0.9 pg/ml). The cut-off value with TBH was 136.3 pg/ml and FNA 63.99 pg/ml. The sensitivity with FNA was lower than those with TBH (χ^2^ = 8.333, P < 0.05) (**Figure 6B**). During the detection process, the TBH method was faster, as the reaction took only 2 min with clear bands; By the FNA method, it took 6 min to complete the reaction. The time consumption of both methods was significantly shorter than that of frozen section examination (> 30 min). Thus, ICGT method can significantly improve the efficiency of intraoperative parathyroid identification.

**Table 1:** Comparison of PTH levels of tissues collected by different sampling techniques.

	FNA	TBH
Non-parathyroid tissues, pg/ml	14.1 ± 0.9	15.3 ± 0.9
Parathyroid gland, pg/ml	224.0 ± 54.6	955.3 ± 16.1
Cut-off value, pg/ml	63.99	136.3
Sensitivity, %	92	100
Specificity, %	100	100
Detection time, min	6	2

### The Diagnostic Accuracy Rate of Immune Colloidal Gold Technique Method in Identifying Parathyroid Tissue

The results of frozen section examination showed 78 parathyroid tissues and 61 non-parathyroid tissues. With biopsy as the standard, the diagnostic accuracy rate was 98.6% (137/139; P > 0.05 vs. biopsy) in the PTH-ICGT group, whereas it was 74.1% in patients identified under direct visual inspection (103/139; P < 0.001 vs. biopsy). Thus, PTH-ICGT exhibited a higher diagnostic accuracy rate (P < 0.001), comparable to the gold standard frozen section examination. Compared with direct visual inspection, ICGT showed a better discrimination capacity than direct visual inspection for identifying both parathyroid glands (97.4 *vs.* 78.2%) and non-parathyroid tissues (100 *vs.* 68.9%; **Figure 6C**). The application of ICGT method can greatly improve the accuracy in identification of parathyroid glands during surgery.

### Part III

#### Clinical Outcomes of Patients Using Parathyroid Hormone-Immune Colloidal Gold Technique Assay in Thyroid Surgery

Of the 106 patients, a total of 111 parathyroid glands were identified and recorded in the control group (n = 53).

**Figure 7:**
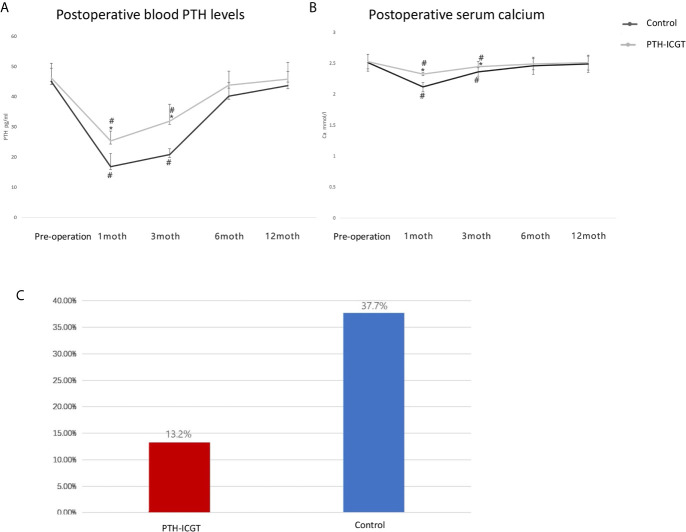
Clinical outcomes of the PTH-ICGT assay in thyroid surgery. The postoperative blood PTH levels **(A)** and serum calcium **(B)** as well as the incidence of postoperative transient hypocalcemia **(C)** were compared between patients who were examined by the PTH-ICGT method and those whose parathyroid glands were identified by the experience of surgeon (control). * shows the difference between the ICGT group and the control group, # shows the comparative difference between preoperative and postoperative within the same group.

The postoperative serum calcium and blood PTH levels as well as the incidence of postoperative transient hypocalcemia were compared between the two groups in order to confirm whether the PTH-ICGT assay could prevent the occurrence of hypoparathyroidism. Patients who were examined by the PTH-ICGT method had higher postoperative serum calcium concentrations and blood PTH levels at 1 and 3 months, as compared with those whose parathyroid glands were identified by the experience of surgeon (P < 0.05, **Figures 7A, B**). However, postoperative serum calcium concentration and blood PTH level became comparable between the two groups of patients at 6 and 12 months (P > 0.05). The incidence of postoperative transient hypocalcemia in the PTH-ICGT group (13.2%, 7/53) was significantly lower than that in the control group (37.7%, 20/53; P < 0.01; **Figure 7C**).

## Discussion

In this study, we introduced a novel technique for distinguishing parathyroid tissue from other structures, a challenge in thyroid or neck surgery. By using an ICGT assay strip with a tissue homogenate sample, ICGT was able to rapidly reflect the PTH concentration in tissue, thus identifying the nature of the tissue and minimizing the probability of parathyroid removal. Given that this technique is rapid, inexpensive, and yields a high diagnostic rate, it has high potential for becoming a novel alternative for intraoperative parathyroid identification.

The use of intraoperative FNA in conjunction with rapid PTH determination has been reported as an alternative to frozen section examination (15). However, this procedure prolongs the surgery time by 40 min and requires special equipment and a trained professional (15). In the present study, a novel assay based on PTH measurement was introduced to shorten the detection time, with a sample measurement read-out time of 2 or 6 min.

Bian et al. applied a Roche ECLIA assay to measure PTH, and found that the mean PTH value was 3,369 pg/ml for determining confirmed parathyroids and was 25.7 pg/ml for determining non-parathyroid tissues, suggesting ECLIA assay as an effective method for differentiating parathyroid and non-parathyroid tissue (12). We first examined the PTH concentrations in 151 serum samples using both the ICGT and ECLIA (Roche) methods. The Passing Bablok analysis revealed good consistency between the PTH measurement results detected by PTH-ICGT and ECLIA, with a correlation coefficient r for consistency of 0.9163. However, the ICGT assay has its own advantages, including easy operation, quick read-out, convenience, and no requirement for expensive equipment and specially trained staff. In addition, ELCIA is only applicable to serum sample detection, whereas the ICGT assay is able to detect PTH levels in both serum and tissue homogenate samples. Thus, these data suggested that the ICGT assay might be served as an alternative method for rapid, intraoperative measurement of PTH concentration, even in tissue homogenate samples.

The intraoperative identification of parathyroid gland tissue by PTH level is dependent on the fact that PTH expression is extremely high only in parathyroid tissue, while it is barely expressed in non-parathyroid tissue, irrespective of the tissue type and location. We found very low levels of PTH in non-parathyroid tissues, such as skeletal muscle, thyroid, fat, and lymph node, suggesting strong tissue-specific expression of PTH. In contrast, PTH concentrations were as high as 955.3 ± 16.1 pg/ml in parathyroid tissue. makes ICGT a reliable method for parathyroid identification by PTH determination.

In this study, we used two methods for sample collection during operation, FNA, TBH. With the different sampling methods, the cut-off values for identifying parathyroid glands varied. FNA had a lower sensitivity compared with TBH. The possible reason may be that the fine needle missed or penetrated the parathyroid tissue, resulting in failure to collect tissue fluid. However, the FNA method has the advantages of convenience, minimal tissue damage, and *in-situ* tissue sampling, and it is recommended for *in-situ* tissue identification. The TBH method is more suitable for *in vitro* tissue identification, with higher sensitivity and specificity as well as property of faster detection.

In this study, the ability of the ICGT assay to discriminate parathyroid glands from non-parathyroid tissue was examined in 106 patients who underwent total thyroidectomy. ICGT showed a higher capacity than direct visual inspection for identifying both parathyroid glands and non-parathyroid tissues. The PTH-ICGT group had a higher postoperative serum calcium concentration and PTH level than the control group at 1 and 3 months. In addition, the PTH-ICGT assay was found to decrease the incidence of transient hypocalcemia, as compared with direct visual inspection by experienced surgeons. The above data reveal that ICGT is a good option for intraoperative parathyroid identification, especially for conditions such as surgery by new surgeons with insufficient experience, second or complicated neck surgery such as thyroid cancer resection, and if the primary hospital is unable to perform frozen section examination or not equipped with expensive equipment.

This study has some limitations. It was a preliminary study performed in a single center with a relatively small sample size. In addition, optimization of this technique, including the optimal cutoff value of PTH, read-out of PTH measurement, and avoidance of false positive/negative detection, is required to realize more accurate and rapid intraoperative identification. These limitations could be overcome by a larger-scale prospective study with a large sample size and multi-center design in the future.

In conclusion, the PTH-ICGT assay has high potential for becoming a novel alternative for intraoperative parathyroid identification based on its advantages including rapid detection with high diagnostic rate. It is particularly applicable in special conditions, such as in surgery performed by new surgeons with insufficient experience, complicated neck surgery, and in the absence of frozen section examination.

## Data Availability Statement

The raw data supporting the conclusions of this article will be made available by the authors, without undue reservation.

## Ethics Statement

The studies involving human participants were reviewed and approved by the ethics committee of Huazhong University of Science and Technology (Wuhan, China). The patients/participants provided their written informed consent to participate in this study.

## Author Contributions

XL, WX, JZ, WS, ZZ, and ZY conceived and designed the research. XL, WX, WS, and ZZ collected the data and conducted the research. JZ and ZY analyzed and interpreted the data. WX, JZ, and ZY wrote the initial paper. WS and XL revised the paper. WX had primary responsibility for the final content. All authors contributed to the article and approved the submitted version.

## Funding

This research was supported by the Clinical Research Physician Program of Tongji Medical College, Huazhong University of Science and Technology (5001540018).

## Conflict of Interest

The authors declare that the research was conducted in the absence of any commercial or financial relationships that could be construed as a potential conflict of interest.

## References

[1] Bilezikian JP Khan A Potts JT Jr. Brandi ML Clarke BL Shoback D. Hypoparathyroidism in the adult: epidemiology, diagnosis, pathophysiology, target-organ involvement, treatment, and challenges for future research. J Bone Miner Res (2011) 26:2317–37. 10.1002/jbmr.483 PMC3405491 21812031

[2] Rubin MR Bilezikian JP. Hypoparathyroidism: clinical features, skeletal microstructure and parathyroid hormone replacement. Arq Bras Endocrinol Metabol (2010) 54:220–6. 10.1590/S0004-27302010000200019 PMC3702727 20485912

[3] Khan MI Waguespack SG Hu MI. Medical management of postsurgical hypoparathyroidism. Endocr Pract (2011) 17 Suppl 1:18–25. 10.4158/EP10302.RA 21134871

[4] De Sanctis V Soliman A Fiscina B. Hypoparathyroidism: from diagnosis to treatment. Curr Opin Endocrinol Diabetes Obes (2012) 19:435–42. 10.1097/MED.0b013e3283591502 23128574

[5] Yu W Zhu L Xu G Song Y Li G Zhang N. Potential role of carbon nanoparticles in protection of parathyroid glands in patients with papillary thyroid cancer. Med (Baltimore) (2016) 95:e5002. 10.1097/MD.0000000000005002 PMC5079313 27759629

[6] Huang K Luo D Huang M Long M Peng X Li H. Protection of parathyroid function using carbon nanoparticles during thyroid surgery. Otolaryngol Head Neck Surg (2013) 149:845–50. 10.1177/0194599813509779 24163324

[7] Patel HP Chadwick DR Harrison BJ Balasubramanian SP. Systematic review of intravenous methylene blue in parathyroid surgery. Br J Surg (2012) 99:1345–51. 10.1002/bjs.8814 22961511

[8] King RC Mills SL Medina JE. Enhanced visualization of parathyroid tissue by infusion of a visible dye conjugated to an antiparathyroid antibody. Head Neck (1999) 21:111–5. 10.1002/(SICI)1097-0347(199903)21:2<111::AID-HED3>3.0.CO;2-Q 10091978

[9] Grubbs EG Mittendorf EA Perrier ND Lee JE. Gamma probe identification of normal parathyroid glands during central neck surgery can facilitate parathyroid preservation. Am J Surg (2008) 196:931–5; discussion 5-6. 10.1016/j.amjsurg.2008.07.026 19095112

[10] Oran E Yetkin G Mihmanli M Celayir F Aygun N Coruh B. The risk of hypocalcemia in patients with parathyroid autotransplantation during thyroidectomy. Ulus Cerrahi Derg (2016) 32:6–10. 10.5152/UCD.2015.3013 26985153 PMC4771430

[11] Takasu H Baba H Inomata N Uchiyama Y Kubota N Kumaki K. The 69-84 amino acid region of the parathyroid hormone molecule is essential for the interaction of the hormone with the binding sites with carboxyl-terminal specificity. Endocrinology (1996) 137:5537–43. 10.1210/endo.137.12.8940381 8940381

[12] Bian XH Li SJ Zhou L Zhang CH Zhang G Fu YT. Applicability of rapid intraoperative parathyroid hormone assay through fine needle aspiration to identify parathyroid tissue in thyroid surgery. Exp Ther Med (2016) 12:4072–6. 10.3892/etm.2016.3896 PMC5228563 28105137

[13] Wang T Sheng S Ruan M Yan J Gu J Jiang Y. Clinical Evaluation of the Immune Colloidal Gold Method for Rapid Qualitative and Quantitative Measurement of Thyroid-Stimulating Hormone as an Assay for Hypothyroidism. Adv Ther (2016) 33:2001–11. 10.1007/s12325-016-0401-y 27605368

[14] Lim V Clarke B. Hypocalcemia. In: Bandeira F, Gharib H, Golbert A, Griz L, Faria M, eds. Endocrinology and Diabetes. New York, NY: Springer (2014). p. 265–78. 10.1007/978-1-4614-8684-8_21

[15] Pelizzo MR Losi A Boschin IM Toniato A Pennelli G Sorgato N. Rapid intraoperative parathyroid hormone assay in fine needle aspiration for differential diagnosis in thyroid and parathyroid surgery. Clin Chem Lab Med (2010) 48:1313–7. 10.1515/CCLM.2010.247 20604733

